# Neonatal Phosphate Nutrition Alters *in Vivo* and *in Vitro* Satellite Cell Activity in Pigs

**DOI:** 10.3390/nu4060436

**Published:** 2012-05-31

**Authors:** Lindsey S. Alexander, Brynn S. Seabolt, Robert P. Rhoads, Chad H. Stahl

**Affiliations:** 1 Laboratory of Developmental Nutrition, Department of Animal Science, North Carolina State University, Raleigh, NC 27695, USA; Email: lindsey.alexander1@gmail.com (L.S.A.); bsseabol@ncsu.edu (B.S.S.); 2 Department of Animal & Poultry Sciences, Virginia Polytechnic Institute and State University, Blacksburg, VA 24061, USA; Email: rhoadsr@vt.edu

**Keywords:** satellite cell, phosphate, neonatal, pig

## Abstract

Satellite cell activity is necessary for postnatal skeletal muscle growth. Severe phosphate (PO_4_) deficiency can alter satellite cell activity, however the role of neonatal PO_4_ nutrition on satellite cell biology remains obscure. Twenty-one piglets (1 day of age, 1.8 ± 0.2 kg BW) were pair-fed liquid diets that were either PO_4_ adequate (0.9% total P), supra-adequate (1.2% total P) in PO_4_ requirement or deficient (0.7% total P) in PO_4_ content for 12 days. Body weight was recorded daily and blood samples collected every 6 days. At day 12, pigs were orally dosed with BrdU and 12 h later, satellite cells were isolated. Satellite cells were also cultured *in vitro* for 7 days to determine if PO_4_ nutrition alters their ability to proceed through their myogenic lineage. Dietary PO_4_ deficiency resulted in reduced (*P* < 0.05) sera PO_4_ and parathyroid hormone (PTH) concentrations, while supra-adequate dietary PO_4_ improved (*P* < 0.05) feed conversion efficiency as compared to the PO_4_ adequate group. *In vivo* satellite cell proliferation was reduced (*P* < 0.05) among the PO_4_ deficient pigs, and these cells had altered *in vitro* expression of markers of myogenic progression. Further work to better understand early nutritional programming of satellite cells and the potential benefits of emphasizing early PO_4_ nutrition for future lean growth potential is warranted.

## 1. Introduction

Although dietary phosphate (PO_4_) is often viewed in the context of its role in bone growth and development, it is also critically important to muscle growth. Postnatal muscle growth has been characterized as a hypertrophic event because fiber numbers are determined during prenatal life and become fixed around the time of birth. However, muscle fibers display a substantial postnatal increase in DNA content, such that between 50–99% of their total DNA is acquired after birth, depending on the species and muscle type [1]. Studies have demonstrated that DNA incorporation precedes the accumulation of muscle protein and that muscle fiber diameter in growing animals is directly related to the total number of myonuclei [1,2,3,4,5,6]. These observations led to the notion that DNA incorporation may be a rate-limiting step for protein accretion and postnatal muscle hypertrophy. Nuclei within muscle fibers are post-mitotic and the postnatal accumulation of DNA is due to the proliferation and fusion of satellite cells to myofibers [7,8]. Therefore, the proliferation and progression of satellite cells through their myogenic lineage is the key factor controlling lifetime muscle growth [2,9,10,11,12]. We have previously demonstrated that in addition to reducing the growth of both muscle and skeletal tissues, severe neonatal dietary PO_4_ deficiency also reduces *in vivo* proliferation of satellite cells [13]. Other neonatal nutrition deficiencies have also been shown to reduce satellite cell activity and subsequent muscle growth [14,15,16,17]. 

While it is intuitive that particular care should be used to avoid nutrient deficiencies in neonates, there has been a historical lack of attention given to dietary PO_4_. The more common concern in regards to PO_4_ nutrition, in both humans and other animals, is the prevention of excessive levels of dietary PO_4_ causing hyperparathyroidism and, in animal agriculture, environmental concerns relating to excess PO_4_ excretion [18,19]. Because neonatal PO_4_ nutrition alters the *in vivo* proliferation of satellite cells [13], and there is support for the nutritional programming of muscle growth via satellite cell activity [14,15,16,17], our objective in this study was to determine the impact of moderate neonatal dietary PO_4_ deficiency and excess on growth, endocrine parameters of PO_4_ homeostasis, and satellite cell activity in the neonatal pig. Characterizing the response of satellite cells to dietary PO_4_ could offer insight into redefining dietary PO_4_ requirements, and into approaches for optimizing lean growth. 

## 2. Experimental Section

### 2.1. Piglets

All animal protocols were approved by the North Carolina State University Institutional Animal Care and Use Committee. Twenty-one cross-bred piglets (24 ± 6 h old, both male and female] were weighed and allotted on the basis of sex and body weight (BW) to 1 of 3 groups which received either a PO_4_ deficient (0.7% total P), adequate (0.9% total P), or a PO_4_ supra-adequate (1.2% total P) milk replacer (Table 1). The Ca content of all diets was adequate (1.2% total Ca). Nutrient requirements for this age of pigs were determined based on the composition of sow’s milk [20] and an extrapolation from NRC requirements for older pigs [21]. All pigs were individually housed in raised cages and fed through a gravity-flow milk delivery system [22]. All piglets were fed equal quantities five times daily and had their total daily intake restricted in order to match the growth rate of sow-reared pigs. Body weight and feed intake were recorded daily throughout the trial. Blood samples were collected initially and every 6 days by venipuncture of the cranial vena cava, and sera was obtained by centrifugation at 3500× *g* and 4 °C. Sera samples were stored at −20 °C until analysis. At the completion of the study, all pigs were orally given 20 mg bromodeoxyuridine (BrdU)/kg body weight in a small quantity of their milk replacer 12 h before tissue collection. Immediately prior to tissue collection pigs were killed by penetrating captive bolt followed by exsanguination. Muscle tissue from the longissimus dorsi was collected aseptically for the isolation of satellite cells and the thyroid was collected for gene expression analysis. Radial bones with attached ulnae were isolated for physical measurements and for determination of mineral content by ashing [13]. 

**Table 1:** Experimental diet composition on an as fed basis ^1^.

	Base Diet ^2,3^
Ingredients	Composition, %
Whey	21.6
Whey protein concentrate	5.0
Edible lard	24.3
Soy protein isolate	30.0
Calcium carbonate	1.2
Dicalcium phosphate	1.0
Calcium chloride	0.33
Mineral premix ^4^	0.8
Vitamin premix ^5^	0.8
Potassium sorbate	0.5
Dextrose	14.6
D, L-Methionine	0.18

^1^ Composition of the powdered milk replacer that was reconstituted at a rate of 175 g/kg final liquid formula; ^2^ Diet was deficient in PO_4_. Potassium phosphate was supplemented to this base diet at reconstitution to create the PO_4_ adequate and supra-adequate diets; ^3^ Manufactured by Milk Specialties Corporation, Dundee, IL; ^4^ Mineral Premix provides per kg: 271 g calcium, 140 mg phosphate, 610 mg sodium, 18.34 g chloride, 129 mg potassium, 14.6 g magnesium, 26.54 g sulfur, 1.85 g copper, 20 g zinc, 68 mg selenium, 124 mg cobalt, 437 mg iodine, 20.8 g iron, 5.44 g manganese, and 60 g choline; ^5^ Vitamin Premix provides per kg: 9.9 g retinyl acetate, 165 mg cholecalciferol, 36.7 mg α-tocopherol, 5.1 g dimethylpyrimidinol bisulfate, 2.04 g thiamin, 8.38 g riboflavin, 4 g pyridoxine, 44 mg vitamin B_12_, 30 g pantothenic acid, 33.1 g niacin, 2.76 g folic acid, 117 g ascorbic acid, and 66 mg biotin.

### 2.2. Sera Analysis

Sera phosphate concentrations were determined by the method of Gomori [23]. Calcium concentrations were determined by flame absorption spectroscopy following dilution in 0.5% lanthium chloride. Sera PTH concentrations were determined using a commercially available kit (Porcine Intact PTH ELISA kit Immutopics, San Clemente, CA).

### 2.3. Isolation of Satellite Cells and Determination of *in Vivo* Proliferation

Satellite cells isolations were from individual pigs according to a procedure modified from Allen *et al*. [24], Rhoads *et al*. [25], and Doumit and Merkel [26]. This procedure varies from our previously reported isolation procedure [13], in that isolations were not pre-plated for 2 h on uncoated plates. Only cell isolations that were greater than 90% PAX7 + were utilized. All cultures were incubated at 37 °C in a humidified environment containing 5% CO_2_. The percentage of cells that had incorporated BrdU into their DNA was determined by immunocytochemistry (anti-BrdU, G_3_G_4_; Developmental Studies Hybridoma Bank, University of Iowa) 24 h after cell isolation [13]. Approximately 200 cells were counted per animal and the percentage proliferation was determined by calculating the ratio of stained cells to total cells. Initial plating densities varied, depending on the number satellite cells isolated from individual animals.

### 2.4. Cell Culture

Cryopreserved satellite cells from individual animals (*n* = 15) from the initial isolation were cultured in proliferation medium (DMEM + 10% FBS + antibiotics) in 15 cm plate, coated with poly-L-lysine and fibronectin until 60% confluence. Cells were then trypsinized and replated in proliferation medium at 2500 cells/cm^2^. Cells were cultured in proliferative medium for 3 days with complete media changes daily. After 3 days of culture, differentiation medium (DMEM + 2% horse serum + antibiotics) was used for an additional 4 days of culture, with complete media changes daily. *In vitro* cell proliferation was determined at day 1 and day 2 of culture using the Click-iT^®^ EdU Alexa Fluor^®^ 488 HCS Assay (Invitrogen, Carlsbad, CA) according to manufacturer’s instructions. Approximately 200 cells were counted per animal and proliferation was measured by calculating the ratio of EdU stained cells to the total number of cells. 

Total RNA was isolated and immunofluorescent staining was performed at 3, 5, and 7 days of culture. Cells were fixed in 2% paraformaldehyde and blocked with 1% bovine serum albumin (BSA) and 0.1% triton X-100 in phosphate buffered saline (PBS). The primary antibodies and their dilutions in PBS containing 1% BSA were as follows: (1) mouse monoclonal anti-PAX7 (1:50; AbD Serotec, Raleigh, NC), (2) rabbit polyclonal anti-MYOD1 (1:100; Santa Cruz Biotechnology, Santa Cruz, CA), and (3) mouse monoclonal anti-MYOG (1:50; AbCam, Cambridge, MA). Secondary antibodies (DyLight 488 AffiniPure donkey anti-mouse IgG and Texas Red AffiniPure goat anti-rabbit IgG, Jackson Immunoreasearch, West Grove, PA) were diluted 1:1000 in PBS containing 1% BSA. Nuclei were stained using 4′,6-diamidino-2-phenylindole (DAPI, Sigma, St. Louis, MO).

### 2.5. Analysis of Gene Expression

Total RNA was isolated from thyroid tissue using RNeasy Midi Kits (Qiagen, Valencia, CA) and from cultured satellite cells using Ambion RNAqueous Micro Kits (Ambion, Austin, TX) according to manufacturer’s instructions. Genomic DNA contamination was removed by treatment with deoxyribonuclease (Ambion DNA free-kit, Austin, TX), and the RNA was then reverse transcribed with Superscript III (Invitrogen Life Technologies, Carlsbad, CA) according to the manufacturer’s instructions. The resulting cDNA samples were then treated with RNase H (Invitrogen, Carlsbad, CA) to ensure the removal of residual RNA. Primer sets (Table 2) were designed using software (Integrated DNA Technologies, Coralville, IA) for the examination of parathyroid hormone (*PTH*), calcitonin (*CALCA*), and calcium sensing receptor (*CASR*) gene expression in thyroid tissue; and for *PAX7*, *MYOD1*, and *MYOG* gene expression in cultured satellite cells. Gene expression was normalized to the expression of two control genes, *RPL35* and *RPL4*, in each sample. Thermocycling conditions included 40 cycles of 20 s of melting at 95 °C followed by 20 s of annealing and extension at 60 °C. Following amplification, all samples were subjected to a melt curve analysis. Gene expression in cultured cells was normalized to the adequate group at day 3 for each gene, using a modification of the 2^−Δ∆CT^ method [27]. 

**Table 2:** Primers used for quantification of gene expression by real-time PCR.

Gene name	Gene ID	Primer sequence
*PAX7*	494466	F: 5′ CAACCACATCCGCCACAAGATAGT 3′
		R: 5′ AGAGGATCTTGGAGACACAGCCAT 3′
*MYOD1*	407604	F: 5′ GCGTGCAAACGCAAGACCACTAA 3′
		R: 5′ AGTCTCGAAGGCCTCGTTGACTTT 3′
*MYOG*	497618	F: 5′ TGACCCTACAGATGCCCACAATCT 3′
		R: 5′ GTTGGGCATGGTTTCATCTGGGAA 3′
*PTH*	399502	F: 5′ ATGCATAACCTGGGCAAACACCTG 3′
		R: 5′ TAGAAGCTCCGAGGGCAACAAAGT 3′
*CALCA*	100579174	F: 5′ TCCAGAGCTAAGCGGTFCAGTAAT 3′
		R: 5′ TTTCTTGCCAGGTGTTTCAGGC 3′
*CASR*	100520980	F: 5′ CATCAAGTTCCGAAACACGCCCAT 3′
		R: 5′ GCAGAGCACGAAGCTAATGCCAAA 3′
*RPL35*	397598	F: 5′ AACCAGACCCAGAAAGAGAAC 3′
		R: 5′ TTCCGCTGCTGCTTCTTG 3′
*RPL4*	6124	F: 5′ CAA GAG TAA CTA CAA CCT TC 3′
		R: 5′ GAA CTC TAC GAT GAA TCT TC 3′

### 2.6. Statistical Analysis

All data was analyzed using the GLM procedure of SAS (Version 9.2, SAS Institute Inc., Cary, NC). Dietary treatment was considered a fixed effect for growth performance, bone, *in vivo* satellite cell proliferation and thyroid gene expression data, and initial BW was used as a covariate for the growth performance data. For the satellite cell culture data, technical replicates were averaged and dietary treatment and time in culture were considered fixed effects. Differences were considered significant at *P* < 0.05.

## 3. Results

Dietary PO_4_ did not have a significant effect on either growth rate or feed intake; however, it did have an effect on feed conversion efficiency (feed intake/BW gain). Pigs that received the supra-adequate PO_4_ diet had improved (*P* < 0.05) feed conversion efficiency compared to those that received the PO_4_ deficient diet. Although pigs fed the PO_4_ adequate diet did not significantly differ from either the PO_4_ deficient or supra-adequate fed pigs, there was an apparent PO_4_ dose response on feed conversion efficiency (Table 3). Similarly, there appeared to be a PO_4_ dose response on the length and mineral content of the radial bones (Table 3), with a trend (*P* < 0.06) for longer bones with higher mineral content among the supra-adequate PO_4_ fed pigs compared to the PO_4_ deficient fed pigs. Pigs fed the PO_4_ deficient diet had wider (*P* < 0.05) radial bones with lower (*P* < 0.05) dry matter percentage than either the adequate or supra-adequate fed pigs. 

**Table 3:** Effect of dietary PO_4_ on growth parameters sera measurements in growing piglets^1^.

	PO_4_ Deficient	PO_4_ Adequate	PO_4_ Supra-adequate
BW gain, g/day	191 ± 5	194 ± 4	201 ± 4
Feed intake, g/day	146 ± 0.7	145 ± 0.6	144 ± 0.6
Feed Efficiency	0.77 ± 0.02 ^a^	0.75 ± 0.01 ^ab^	0.72 ± 0.01 ^b^
Sera Ca, mM			
day 6	2.91 ± 0.13 ^a^	2.48 ± 0.13 ^b^	2.57 ± 0.13 ^ab^
day 12	2.80 ± 0.14	2.71 ± 0.14	2.61 ± 0.14
Sera PO4, mM			
day 6	1.70 ± 0.06 ^b^	1.98 ± 0.06 ^a^	2.05 ± 0.06 ^a^
day 12	1.62 ± 0.08 ^b^	2.17 ± 0.08 ^a^	2.26 ± 0.08 ^a^
Sera PTH, pg/mL			
day 6	1.5 ± 2.6 ^b^	13.6 ± 2.6 ^a^	15.9 ± 2.6 ^a^
day 12	5.0 ± 15.1 ^b^	37.6 ± 15.1 ^ab^	52.0 ± 15.1 ^a^
Bone mineral content, g	1.61 ± 0.08	1.72 ± 0.08	1.84 ± 0.08
Bone Ash %	32.48 ± 1.02	34.02 ± 1.02	34.16 ± 1.02
Radial Length, mm	7.12 ± 0.10 ^b^	7.25 ± 0.10 ^ab^	7.42 ± 0.10 ^a^
Radial Width, mm	0.94 ± 0.03 ^a^	0.81 ± 0.03 ^b^	0.84 ± 0.03 ^b^
BrdU + Satellite Cell, %	9.9 ± 1.4 ^b^	15.6 ± 1.5 ^a^	17.6 ± 1.7 ^a^

^1^ Both male and female pigs were utilized, PO_4_ deficient and PO_4_ supra-adequate (*n* = 7, 5 males and 2 females) and PO_4_ adequate (*n* = 7, 4 males and 3 females); ^a,b^ Values within a row not sharing a common superscript are different (*P* < 0.05).

Pigs fed the PO_4_ deficient diet had reduced (*P* < 0.05) sera PO_4_ and PTH and elevated (*P* < 0.05) sera Ca levels at day 6 (Table 3). At day 1–2 on study, the effect of the PO_4_ deficient diet on sera PO_4_ remained however, sera Ca levels were no longer significantly different among the treatment groups. Additionally at the completion of the feeding trial, the pigs receiving the PO_4_ deficient diet had approximately 10 fold lower (*P* < 0.05) sera PTH concentrations than pigs receiving the PO_4_ supra-adequate diet. While the PTH concentrations of the pigs fed the PO_4_ adequate diet were not significantly different from those of pigs fed the other two diets, an apparent dietary PO_4_ dose dependent response in sera PTH concentrations was seen (5.0, 37.6, and 52.0 pg/mL for deficient, adequate, and supra-adequate respectively).

**Figure 1:**
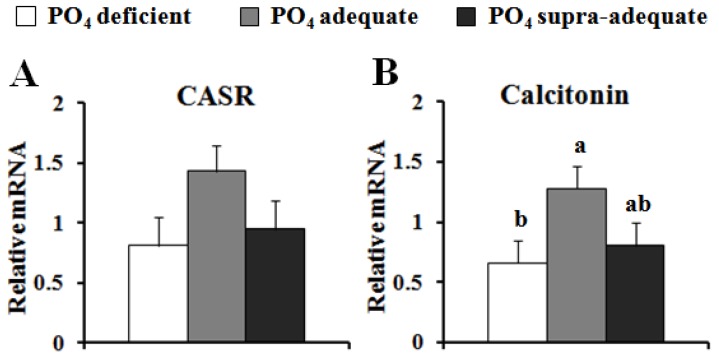
Effect of dietary PO_4_ on the gene expression of (**A**) CASR, and (**B**) calcitonin, in the thyroid of neonatal pigs. Values presented are least square means and standard error of values normalized to cDNA concentrations (*n* = 7). ^a,b^ Values not sharing a common superscript are different (*P* < 0.05).

There was reduced (*P* < 0.05) *in vivo* proliferation of satellite cells isolated from pigs fed the PO_4_ deficient diet compared to all other pigs (Table 3). There was no statistically significant difference in the percentage of BrdU labeled cells isolated from pigs fed the PO_4_ supra-adequate diet when compared to the PO_4_ adequate group. There were no significant differences in the gene expression of PTH in thyroid tissue among the treatment groups. There was greater *CALCA* gene expression in the PO_4_ adequate group compared to both the PO_4_ deficient and the PO_4_ supra-adequate groups (*P* < 0.05 and *P* < 0.08, respectively) (Figure 1). A similar, but not statistically significant (*P* < 0.06 and *P* < 0.13, respectively), pattern of gene expression in the thyroid was seen for *CASR*. 

**Figure 2:**
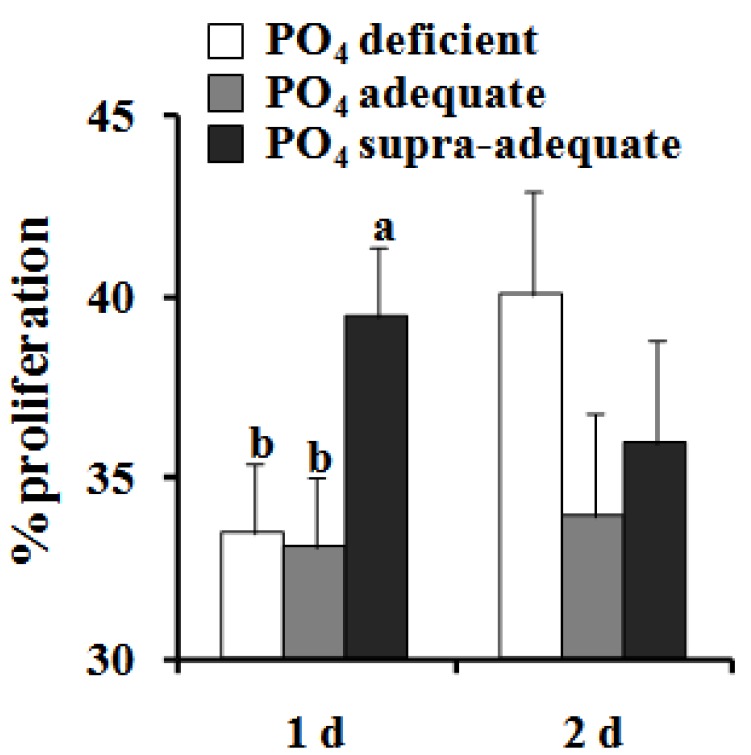
Effect of dietary PO_4_ on *in vitro* satellite cell proliferation. ^a,b^ Values not sharing a common superscript are different (*P* < 0.05). Values presented are least square means and standard error (*n* = 7).

**Figure 3:**
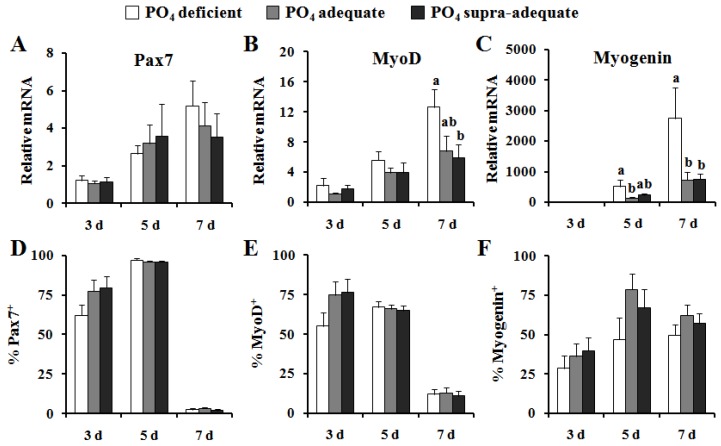
Effect of dietary PO_4_ on the gene expression (top panels) and *in vitro* protein (bottom panels) of myogenic regulatory factors: (**A**, **D**), Pax7, (**B**, **E**) MyoD, and (**C**, **F**) myogenin in satellite cells isolated from neonatal pigs. Values presented are least square means and standard error of values; either percentage of stained nuclei or normalized to cDNA concentrations (*n* = 7). ^a,b^ Values within a timepoint not sharing a common superscript are different (*P* < 0.05).

Greater proliferation (*P* < 0.05) was observed in cells isolated from pigs receiving supra-adequate dietary PO_4_ 1 day after plating relative to cells isolated from pigs in the other treatment groups (Figure 2). After 2 days of culture in proliferative medium, dietary treatment no longer affected proliferation rate. Gene expression of *PAX7* increased over time but did not differ between treatment groups at any time point. The expression of *MYOD1* mRNA also increased overtime, and the levels at day 7 were approximately 2 fold greater (*P* < 0.05, Figure 3B) in cells isolated from PO_4_ deficient pigs compared to those isolated from pigs receiving supra-adequate dietary PO_4_. The *MYOD1* gene expression levels seen in cells isolated from the PO_4_ adequate pigs were intermediate to, and not statistically different from, either of the other treatment groups. A similar pattern was observed in *MYOG* gene expression (Figure 3C). After 5 days of culture, *MYOG* expression in PO_4_ deficient cells was 3-fold higher (*P* < 0.05) than PO_4_ adequate cells and tended to be higher than PO_4_ excess cells (*P* = 0.1). After 7 days of culture, *MYOG* expression in satellite cells isolated from PO_4_ deficient pigs was 3-fold higher (*P* < 0.05) than in cells isolated from either pigs fed the PO_4_ adequate or the supra-adequate diets (Figure 3C).

Immunofluorescent staining was performed to complement our analysis of changes in gene expression, and is reported as a percentage of positively stained nuclei. After 3 days of culture, satellite cells from both PO_4_ adequate (*P* = 0.14) and PO_4_ supra-adequate (*P* = 0.1) pigs tended to have a higher percentage of PAX7^+^ nuclei (Figure 3). The percentage of PAX7^+^ cells increased to over 95% in all treatment groups by day 5 of culture, and decreased to less than 4% in all treatment groups by day 7 (Figure 3). Cells from PO_4_ deficient pigs tended to have a lower percentage of MYOD1^+^ cells relative to PO_4_ adequate (*P* < 0.13) and PO_4_ supra-adequate (*P* < 0.1) cells at day 3 of culture (Figure 3). Although there were no differences in MYOD1 staining among treatment groups at day 5, it should be noted that the percentage of MYOD1^+^ cells increased in cells isolated from PO_4_ deficient pigs while percentages decreased in cells from both PO_4_ adequate and PO_4_ supra-adequate fed pigs. The percentage of MYOD1^+^ cells decreased dramatically in all groups by day 7 of culture. There were no significant differences in MYOG staining among the treatment groups. The percentage of MYOG positive nuclei doubled between day 3 and day 5 of culture and remained elevated at day 7. 

## 4. Discussion

In this study, we evaluated a range of dietary PO_4_ concentrations fed to neonatal pigs and characterized their response on both a whole animal and cellular level. Since we had previously demonstrated that a severe neonatal PO_4_ deficiency dramatically impacted growth, satellite cell proliferation, and endocrine parameters of PO_4_ status [13], we wanted to examine a more realistic range of dietary PO_4_ levels to neonatal pigs ranging from a subclinical deficiency to a marginal excess. We achieved a subclinical dietary PO_4_ deficiency among the piglets fed the PO_4_ deficient diet as evident by reduced sera inorganic PO_4_ and PTH, without a reduction in growth rate and only marginal effects on bone growth. We and others have previously demonstrated reduced PTH in response to dietary PO_4_ deficiency, independent of either dietary or circulating levels of Ca [13,28]. Despite being of tremendous importance to the homeostatic regulation of both Ca and PO_4_, the interplay between PTH and 1,25-dihydroxycholecalciferol is absent in the neonate. The 1,25-dihydroxycholecalciferol regulatory system is undeveloped or inactive in the neonates [13,29,30,31]. Therefore, it appears that PTH is the major regulatory hormone of mineral homeostasis in neonates. 

Neonatal nutrient deficiencies have been shown to reduce satellite cell number and activity and lead to permanent muscle growth deficits [14,32]. While nutrient restriction negatively impacts the activity of satellite cells, the stimulatory effect of nutrition, via insulin signaling and/or AMPK and mTOR signaling, on neonatal muscle growth may also be mediated through satellite cells [33,34]. These works provide support for the critical nature of early life nutrition on lifetime growth. While the need to avoid extremes of both dietary PO_4_ deficiency and excess in neonates is intuitive, very little research has been conducted to examine the role of dietary PO_4_ concentrations over the subclinical range in neonates. The potential for lifetime impact on muscle growth via alterations in satellite cell activity coupled with the requirement of dietary PO_4_ for muscle growth and its effect on satellite cell activity *in vivo* [13], necessitated further examination of the role of dietary PO_4_ on satellite cell activity. Interestingly, despite the subclinical nature of both the dietary PO_4_ deficiency and excess, both diets had a significant impact on satellite cell activity. The subclinical PO_4_ deficiency that was generated in our study resulted in reduced *in vivo* proliferation of satellite cells. As expected, the reduction in the *in vivo* proliferation of satellite cells seen in this study was less dramatic than what was seen previously with a severe PO_4_ deficiency (36.5% *vs.* 70% reduction in proliferation) [13]. When cultured *in vitro*, the proliferation rate of satellite cells from the PO_4_ deficient pigs did not differ from those of the PO_4_ adequate pigs. Although there was not a significant difference within sampling time point, it is interesting to note that the rate of proliferation of satellite cells from the PO_4_ deficient pigs experienced a dramatic increase on day 2 of culture compared to day 1. This increase in proliferation could be a compensatory response to the greater PO_4_ content of the proliferation medium. A similar increase in the *in vitro* satellite cell proliferation rate of satellite cells obtained from feed-restricted turkey poults has been reported [16]. Satellite cells isolated from the PO_4_ deficient pigs also had altered gene expression of the myogenic regulatory factors *MYOD1* and *MYOG* as compared to the cells isolated from PO_4_ adequate pigs. The greater gene expression of *MYOD1* and *MYOG* seen among the PO_4_ deficient satellite cells did not correspond to an increase in the percentage of cells that stained positive for the corresponding proteins. In fact, there was actually a trend for reduced MYOD1 staining at day 3 among PO_4_ deficient satellite cells compared to the adequate group. The increases in *MYOD1* and *MYOG* gene expression seen among satellite cells isolated from PO_4_ deficient pigs in this study, do not appear to correspond to greater myogenic differentiation. Based on reduced muscle hypertrophy caused by severe dietary PO_4_ deficiency, it is intriguing to hypothesize that the elevated *MYOD1* and *MYOG* gene expression may be linked to increased apoptotic regulation in these cells. The importance of MYOD1 in regulating the apoptosis of myoblasts via inducing the expression of miR-1 and miR-206 has been demonstrated [35,36]. 

The activity of satellite cells isolated from the pigs receiving the PO_4_ supra-adequate diet were also altered compared with the cells isolated from the PO_4_ adequate pigs. While not statistically significant, there was a numerical increase in the number of proliferating satellite cells *in vivo*. When cultured *in vitro*, the satellite cells isolated from the PO_4_ supra-adequate fed pigs had a significantly greater (*P* < 0.05) proliferation rate compared to the PO_4_ adequate cells. This, coupled with the numerical increase in the *in vivo* proliferation rate suggests that excess PO_4_ nutrition may increase the proliferative capacity of satellite cells. There were no differences in the gene expression of the myogenic regulatory factors examined or in the staining for these proteins between the cells isolated from the supra-adequate or the adequate PO_4_ fed pigs. Although there was not a statistically significant difference in growth rate among the treatment groups, the supra-adequate PO_4_ fed pigs tended to have greater BW gain than both the deficient and the adequate PO_4_ fed groups (*P* < 0.10 and *P* < 0.17, respectively). The slight increase in BW gain coupled with equal feed intake resulted in the pigs fed the PO_4_ supra-adequate diet having an improved feed conversion efficiency when compared to the other treatment groups (*P* < 0.03 and *P* < 0.16 for the comparisons with the PO_4_ deficient and the adequate, respectively). When viewed together, the changes seen in satellite cell activity, the tendency for improved growth and improved feed conversion efficiency are suggestive that supra-adequate PO_4_ nutrition increases muscle growth in neonatal pigs. 

## 5. Conclusions

In this study, we have demonstrated that early neonatal PO_4_ nutrition impacts satellite cell activity both *in vivo* and *in vitro*. If the altered *in vitro* activity is truly indicative of a programming event, this could have lifetime consequences for muscle growth and development because the proliferation and myogenic progression of satellite cells is the key factor controlling lifetime muscle growth [2,9,10,11,12]. Of particular importance in this study is that alterations in satellite cell activity were seen with a subclinical dietary PO_4_ deficiency. This stresses the importance of understanding how dietary PO_4_ influences developmental programming of muscle tissue, particularly in situations of potentially compromised PO_4_ status (*i.e.*, premature or small for gestational age humans and for low birth-weight piglets). Reductions in lean growth potential could lead to increased risk of obesity in humans and would have substantial economic and environmental consequences for commercial swine production. It is evident that further research is needed to define neonatal nutrient requirements in the context of the potential of nutritional programming of growth.
